# Spotted Lanternflies Respond to Natural Pheromone Lures for Mate-Finding and Oviposition

**DOI:** 10.3390/insects15060447

**Published:** 2024-06-13

**Authors:** Miriam F. Cooperband, Kelly M. Murman

**Affiliations:** Forest Pest Methods Laboratory, USDA—APHIS—PPQ, 1398 W. Truck Rd., Buzzards Bay, MA 02542, USA

**Keywords:** traps, lures, honeydew, insect communication, pheromones, synergism, aggregation, *Lycorma delicatula*, mate-finding, oviposition

## Abstract

**Simple Summary:**

The spotted lanternfly (SLF) is an invasive generalist that is spreading throughout the eastern United States. They feed in large aggregations, but the mechanisms they use to form these aggregations and find each other for mating are poorly understood. Laboratory evidence that they use pheromones is based on volatiles from their bodies and from honeydew excretions, but, due to their broad sensory capabilities, the pheromone components have been difficult to isolate and identify. This is the first evidence gathered in a field study that demonstrates, using naturally collected SLF odors as lures, that volatiles from SLF bodies and honeydew are both required to attract males at mating time and females during oviposition time.

**Abstract:**

Using semiochemicals collected from spotted lanternflies *Lycorma delicatula* (Hemiptera: Fulgoridae) (SLF) and deployed in the field with circle traps, we demonstrated that SLF responded to SLF pheromones: in particular, this was the case for males while seeking mates and for females while ovipositing. The attractants consisted of SLF body extract emitted from diffuser lures and SLF honeydew on burlap ribbons, collected from heavily infested locations. Traps with attractants were deployed in field sites with very light SLF infestations to avoid competing signals of pre-existing aggregations. The number of SLF equivalents emitted by each diffuser per trapping period was used in a dose–response analysis. Three trees per block received either (1) a control hexane lure and a clean ribbon, (2) a lure containing SLF extract and a clean ribbon, or (3) a lure containing SLF extract and a honeydew-laden ribbon. Ten blocks were sampled three times per week for twelve weeks. We found a significant positive dose–response by males to SLF body extract only in the presence of SLF honeydew, indicating a synergistic effect between honeydew volatiles and body volatiles. This dose–response occurred for five weeks after mating started, after which males no longer responded. Subsequently, females had a significant positive dose–response to SLF extract only in the presence of honeydew when oviposition was their primary activity, continuing for two weeks, suggesting that females may use pheromones to aggregate for oviposition. The extract in the absence of honeydew did not result in a positive dose–response, nor did the hexane control. These findings suggest that SLF respond synergistically to the combination of pheromones present in both SLF honeydew and SLF bodies. Thus, combining key components from both sources may aid the development of semiochemical lures for SLF.

## 1. Introduction

The spotted lanternfly (SLF), *Lycorma delicatula* (White) (Hemiptera: Fulgoridae), is an invasive planthopper that feeds on the phloem of over 100 species of plants [1,2]. Although a generalist, their preferred host is *Ailanthus altissima* (Mill.) Swingle (Sapindales: Simaroubaceae), for which they gradually narrow their host preference as they develop into adulthood [3]. They engage in aggregation behavior as nymphs and adults, spanning times when their primary activities are feeding, mating, and oviposition [4,5]. These aggregations can directly cause serious problems by depleting plant resources and reducing their ability to generate new growth or overwinter [1]. In this way, they have caused economic damage to vineyards [6]. Indirect damage to plants also occurs when honeydew, excreted while feeding, coats foliage and fosters sooty mold growth, blocking photosynthesis and killing understory plants [6]. In addition, large aggregations of SLF become nuisance pests when copious amounts of sugary honeydew and sooty mold coat outdoor furniture, decks, and vehicles and attract other insects like bees, wasps, and ants, interfering with outdoor enjoyment [6].

How SLF locate each other for aggregation and mating remains somewhat of a mystery, but the available evidence currently suggests that they utilize a combination of plant-derived cues as well as multimodal intraspecific communication. Specifically, there is a growing body of evidence to suggest that, in addition to using host plant odors (kairomones) [7,8], they emit species-specific chemical signals (pheromones) as well as mechanical signals (substrate vibrations) (pers. obs., Cooperband). However, the specific chemical and vibroacoustic signals responsible for eliciting specific behaviors have not yet been identified and described. An array of 78 volatile semiochemicals have been identified for SLF so far, 24 of which are associated with plants [7,8,9,10], 13 were identified from their honeydew excretions [11], and 44 were derived from SLF bodies themselves [12,13,14], with 3 compounds sharing more than one source. The honeydew and body odors appear to contain sex-specific attractive pheromones based on olfactometer responses, but the specific pheromone components have not yet been determined. Additionally, there is evidence that SLF communicate through substrate vibrations (pers. obs., Cooperband) [15].

We are beginning to understand the mechanisms by which SLF detect semiochemicals. Chemosensory organs located primarily on their antennae [14,16] and on their proboscis [17,18] are responsible for sensing a range of semiochemicals. The antennae of SLF can detect volatile components identified from plants, SLF honeydew, and SLF bodies, with the antennae being much more sensitive to some compounds than others [14]. Adult male antennae are most sensitive to compounds from honeydew, whereas adult female antennae are most sensitive to different compounds.

An important step in mitigation is early detection, using traps and lures to detect new pest populations. Although efficacious traps have been developed for SLF relying upon the negative gravitaxis of SLF walking up tree trunks, including inverted sticky bands and circle trunk traps which guide SLF into a collection bag or jar with a kill strip [19,20,21], currently, the only lure reported to improve trap capture when placed on a host plant is a high-release methyl salicylate lure, which attracts SLF of all stages [5,7,8]. However, this lure alone cannot outcompete the natural signals produced when SLF aggregate [5].

The slow progress in lure development is a result of several challenges. One is the large number of antennally and behaviorally active semiochemicals known for SLF, which leads to an even larger number of possible blends, ratios, and release rates. Since 2015, we have conducted over 50 field tests using different sets of compounds in a variety of blends and ratios, but, so far, none has resulted in significant attraction in the field (Cooperband, unpublished). The recent comparison of male and female SLF antennal sensitivities revealed sexually dimorphic responses to certain SLF-derived compounds, which may be helpful in targeting compounds which elicit different behaviors in males and females [14]. Additionally, in comparing the volatile profiles of males and females, few obvious sexual differences in the presence or absence of compounds have been reported [11,13]. If a key component is not included, no amount of adjusting ratios and release rates for blends without it would improve attraction to lures. While investigations continue to decipher which compounds are critical in SLF communication, we also continue to gather behavioral information to assist in this effort.

Progress in understanding how SLF locate one another for aggregation and mating has also been gradual, as is necessarily the case with univoltine insects which feed on large trees and cannot practically be reared. Challenges remain in collecting and identifying SLF pheromones, the compounds responsible for aggregation and mate-finding. Although we have demonstrated that we can stimulate aggregation behavior in the field using living SLF enclosed in sleeves on trees as lures [4], the resulting aggregation response does not rule out attraction to plant damage volatiles or substrate vibrations produced by the living caged SLF, nor does it separate responses to honeydew volatiles from those to pheromones emitted by the SLF themselves. Although we have evidence of pheromone use by SLF, as demonstrated by SLF’s responses to their own odors in behavioral bioassays in the laboratory, such responses have not been reproduced in the field using synthetic lures composed of the known semiochemicals. Further, when using a dual-choice olfactometer comparing synthetic semiochemicals to a blank control, it is difficult to discern between attractive compounds of varying importance; thus, field testing is crucial to SLF lure development. If we can attract SLF to a natural pheromone lure in the field, researchers can be encouraged to continue efforts towards identifying the pheromone components and developing them into a synthetic lure.

The current study tested naturally occurring SLF in the field for attraction to their naturally produced and collected odors. By doing this, we excluded attraction due to host plant damage and substrate vibrations and focused only on pheromones, with assurance that all the key components, even those which we may not know about yet, were present in the lure and in the correct ratios.

## 2. Materials and Methods

### 2.1. Field Sites and Experimental Design

This field study was conducted in 2022 to test whether the naturally collected volatiles from honeydew and whole SLF bodies could be used to attract SLF in the field. The field sites consisted of forested areas containing their preferred host, mature *A. altissima* trees, on private, industrial, and public properties where no pesticides were being used. Permission was obtained from the property owners and managers to conduct our work.

**Figure 1:**
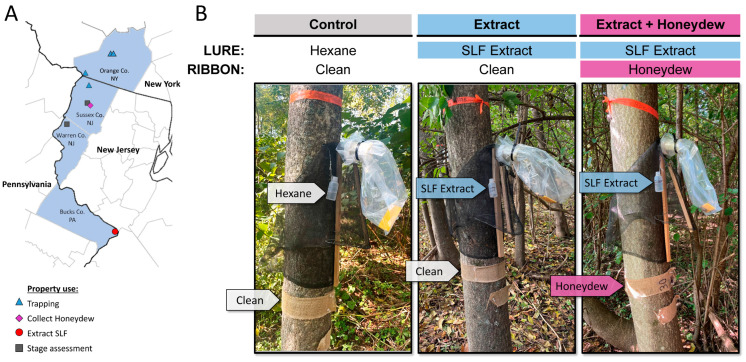
(**A**) A map with the counties (shaded blue) where the activities took place, such as trapping, collecting honeydew, extracting spotted lanternflies *L. delicatula* (SLF), and SLF stage assessment, depicting the approximate locations and usage of properties. (**B**) The three treatments are depicted on *A. altissima* trees fitted with circle traps and plastic collection bags. The “*Control*” had a diffuser bottle containing only hexane and a clean burlap ribbon. The “*Extract*” had a diffuser bottle containing whole-body extract of SLF and a clean burlap ribbon. The “*Extract* + *Honeydew*” had a diffuser bottle containing whole-body extract of SLF and a burlap ribbon laden with fresh SLF honeydew. (Photo credits: A. Greenwood, K. Kaye, and M. Cooperband).

To reduce interference by the signals produced from naturally occurring SLF in the field, only extremely low-SLF-density field sites were used as the study blocks. One study block was located on a property in Sussex County, New Jersey, and three properties containing two, three, and four study blocks, respectively, were located in Orange County, New York (Figure 1A).

High-density field sites, used for making SLF extract and collecting SLF honeydew, were located in Bucks County, Pennsylvania, and Sussex County, NJ, respectively (Figure 1A). The natural odors collected from high-density sites were deployed as lures at low-density sites every Monday, Wednesday, and Friday, for 12 weeks. Trapping commenced on the 8th of August and was concluded on the 31st of October 2022.

The timing of adult stages was determined based on daily observations in Sussex County and Warren County, New Jersey, between the high- and low-density field sites used in this study followed previously published protocols [4] (Figure 1A). When SLF emerge as adults, for the first few weeks, they are in the Early-1 stage, when feeding is the main activity observed. Early-2 is marked by an abrupt shift in the sex ratio observed on large *A. altissima* trunks, causing males to appear scarce [22]. During this Early-2 stage, feeding is still the main activity observed, but large aggregations of mostly females form on *A. altissima* trees. The Mid stage starts with the first observation of mating in the field. Late-1 starts with the first observation of a freshly deposited egg mass in the field. Late-2 starts two weeks after Late-1. Late-3 starts two weeks after Late-2 and ends when adult SLF die off for the season, usually around the first week of November or when the first hard frost occurs.

Ten blocks of study trees were selected for trapping, with each block containing three *A. altissima* trees with an average diameter at breast height (DBH) ± SE of 18.5 ± 0.5 cm. The average DBH difference between the largest and smallest trees in the same block was 2.8 ± 0.4 cm, and the difference was always less than 5 cm. Trees in the same block were spaced 3–7 m apart, and the blocks were at least 15 m apart. Traps were set at each site in advance of this study to survey the nymphal SLF population, ensuring that these sites had extremely low-density populations of SLF. This was typically where the leading edge of the invasive SLF population had just arrived. Each tree was fixed with a circle trunk trap for SLF and a plastic collection bag (GL/GL-4011-00, Great Lakes Entomology, Vestaburg, MI, USA) containing an insecticidal pest strip (Vapona II, 2,2-dichlorovinyl dimethyl phosphate (10%), Hercon Environmental, Emingsville, PA, USA) to kill the captured SLF [19,20].

The three treatments consisted of (1) a control lure containing 30 mL of hexane and a clean burlap ribbon, (2) a lure containing 30 mL of SLF whole-body extract and a clean burlap ribbon, and (3) a lure containing 30 mL of SLF whole-body extract and a burlap ribbon with SLF honeydew on it (Figure 1B). The burlap ribbons and lures are further described in the sections below.

Each trap was serviced three times per week for 12 weeks. Thus, a “trapping period” referred to the time between Mondays, Wednesdays, and Fridays (to accommodate for holidays, a few trapping periods were adjusted by one day). For trap servicing, the lure was changed, the honeydew-laden burlap ribbon was changed, and the bag containing trapped SLF was removed and replaced with an empty bag with a pest strip.

### 2.2. Burlap Ribbons Laden with Crude Honeydew

Burlap ribbons (6.7 cm wide, T-BurlapRibbon-3inch, Tosnail, China) cut to approximately 78 cm in length were secured around the tree trunk below the trap. Each “Control” and “Extract” tree received a clean burlap ribbon, whereas each tree assigned with the “Extract + Honeydew” treatment received a burlap ribbon laden with a fresh accumulation of honeydew at the beginning of each trapping period (Figure 1).

For the “Extract + Honeydew” treatment, the burlap ribbon first was brought to a high-SLF-density site and suspended between two heavily infested trees where honeydew was observed to be falling and coating the understory. Using push pins, the ribbon was attached at both ends to adjacent *A. altissima* trees in such a manner that its surface was exposed horizontally to catch the falling honeydew droplets. These burlap ribbons were left to collect honeydew for 2–3 days and then transported to the study blocks, where they were attached under the traps on the trees receiving the “Extract + Honeydew” treatment. The schedule for collecting honeydew on these burlap ribbons was as follows: one set of ten ribbons collected honeydew from Mondays to Wednesdays and was set with extract lures from Wednesdays to Fridays; a second set of ten ribbons collected honeydew from Wednesdays to Fridays and was set with extract lures from Fridays to Mondays; and a third set of ribbons collected honeydew from Fridays to Mondays and was set with lures from Mondays to Wednesdays. These same 30 honeydew-laden ribbons were reused weekly throughout the season, and the block assignments for these ribbons were randomized such that they were in a different block each week.

### 2.3. Extract Diffusers

The lures consisted of applicator bottles (30 mL, LDPE, BENECREAT™, Shenzhen Junxin Technology Co., Ltd., Shenzhen, China) crafted into diffusers by adding a wick that extended from the lid. The wicks were 2 mm diameter × 15 cm natural twisted cotton strings extending from within the bottle to outside the applicator tip. A knot was used to secure the position of the wick extending from the applicator tip. The exposed wick ranged from 1.5 to 6 cm and was adjusted in each trapping period to aim for a steady evaporation rate of all the contents over the trapping period. The wick length was based on how much extract remained in the diffusers in the previous trapping period, and, in colder weather in the fall, the wick and the cap had to be removed, using an open bottle to approach the desired diffusion rate.

At the beginning of each trapping period, the bottles were filled with either 30 mL of SLF whole-body extract in hexane that had been made on the preceding Thursday (see below) or 30 mL of hexane (control). The diffuser lures were positioned inside the trap opening on the north side of the tree to reduce variation in the release rate due to sun exposure.

At the end of every trapping period, before each diffuser was refilled, the contents remaining in the diffuser were measured to determine the evaporation rate for that trapping period. That remaining extract was then deposited onto a piece of burlap ribbon attached to the tree trunk below the trap and immediately evaporated prior to refilling the diffusers with fresh extract.

The volume and concentration of the extract that evaporated from each deployment until the subsequent trap servicing were used to calculate the number of SLF equivalents per day emitted from each lure during each trapping period. For the controls, the volume of hexane that evaporated was similarly multiplied by the hypothetical SLF concentration used for the extract treatments to compare the evaporation rates of the hexane controls with the extracts.

### 2.4. SLF Whole-Body Extract

**Figure 2:**
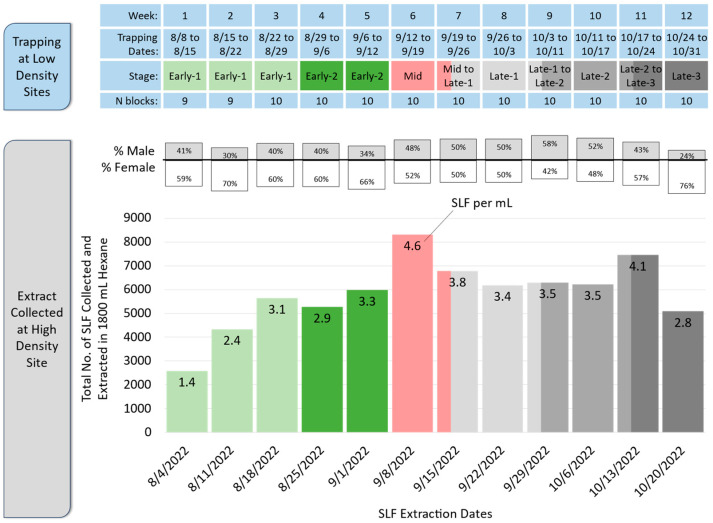
The trapping dates and stages of *L. delicatula* spotted lanternflies (SLF) at low-density sites (**top**), and the sex ratios, numbers, and dates of the extracted SLF and the number of SLF equivalents per mL for each extraction date, conducted at the high-density site (**bottom**).

Every Thursday, enough SLF whole-body extract was made to load the lures on the following Monday, Wednesday, and Friday. The goal each Thursday was to collect and extract as many SLF as possible and then generate 1800 mL of extract, with a target minimum concentration of 1 SLF equivalent per ml. This goal was exceeded every week, with weekly extracts which ranged from 1.4 to 4.6 SLF equivalents per ml (Figure 2).

On Thursdays, from the 4th of August until the 20th of October 2022, four-to-eight technicians travelled to a heavily infested field site in Morrisville, Pennsylvania, where they spent the day collecting and extracting several thousand SLF as they removed them from *A. altissima* tree trunks. Nitrile gloves and eye protection were worn. Each technician carried a 600 mL stainless steel container with a flip lid. Each container received 300 mL of *n*-hexane (Thermo Fisher Scientific, Fair Lawn, NJ, USA). Live SLF were removed from the trees and placed directly into the hexane while being counted. After approximately 150 SLF had been collected and extracted in this way, which took approximately 20–25 min, the extracted SLF were removed by decanting the extract through a stainless steel strainer into another stainless steel container. The extract was then used for the continued collecting and extracting of more SLF. This was repeated three or more times, totaling 450 or more SLF extracted into each 300 mL of hexane. The extracted SLF bodies that had been strained out were air-dried, 100 of them were sexed to determine their sex ratio, and then they were discarded. When collecting was finished, the extract volume was measured and brought up to 1800 mL by adding hexane, then poured into an empty hexane bottle and transported to an explosion-proof freezer (−20 °C), where it was stored until use. The total number of SLF extracted and the volume of extract generated each Thursday were used to calculate SLF equivalents per ml for each batch of extract.

The lure diffuser bottles received 30 mL of extract on the Monday, Wednesday, and Friday following the Thursday on which it had been made. It was noted that the extracted volatiles were deployed nearly a week after they had been collected, but it was also noted that the site where the extracts were being made was nearly a week ahead in terms of SLF development compared to where the lures were being deployed due to differences in latitude. Therefore, the differences in timing would cancel each other out.

### 2.5. Statistical Analysis

The SLF captured by each lure and trap in each trapping period were frozen, sexed, and counted. For each lure and trapping period, the number of SLF equivalents per ml of extract was multiplied by the amount of extract emitted by that lure and divided by the number of days in that trapping period to give the number of SLF equivalents emitted per day, which was plotted against the lure’s trap catch.

All data were analyzed using JMP (v10.0.0, SAS Institute Inc., Cary, NC, USA). Four models were compared to determine the best fitting model to use for the dose–response analysis, and a linear regression model was a better fit than a three-, four-, or five-parameter logistic model. For each extract date, a linear regression analysis was conducted separately on the number of males and females captured per trapping period compared to the number of SLF equivalents emitted per day by each corresponding lure during that trapping period. A significant dose–response occurred when *p* < 0.05.

## 3. Results

The range of SLF equivalents emitted per day for the control (hypothetical SLF equivalents), extract, and extract + honeydew treatments, respectively, was 6.6 to 56.5, 3.8 to 46.9, and 4.8 to 45.4. The average number of SLF equivalents emitted per day for the entire experiment was 21.6 ± 0.54, 21.0 ± 0.45, and 21.1 ± 0.42 for the respective treatments.

One field site had to be replaced after the first two weeks due to access problems, so it was omitted from our analysis, resulting in N = 9 for weeks 1 and 2, and N = 10 for the remaining weeks. During those first two weeks at the very low-density field trapping sites, the traps were mostly empty; so, bags in weeks 1 and 2 were replaced only once per week. Starting in week 3, there were three trapping periods per week, with the same batch of extract used for the three trapping periods in a week; thus, there were thirty data points for each treatment per extract week. Until weeks 4 and 5, respectively, too few female or male SLF were captured to compare the fits of the different regression models. Of the remaining weeks for females and males, the majority of the weekly data fit best with a linear regression model, with average AICc [23,24] weights for the linear, three-parameter logistic, and four-parameter logistic models, respectively, being 0.71 (±0.08), 0.22 (±0.06), and 0.07 (±0.04), and no data fit the five-parameter logistic model. Therefore, a linear regression analysis was conducted on all the data.

No significant relationships were found between the amount of hexane emitted from the control lures per day and the number of SLF of either sex caught over the entire 12-week study (Figure S1). There were also no significant relationships found between SLF caught and the amount of SLF equivalents emitted from the extract in the remaining two treatments in the first five weeks of this study (Figure S2). For the extract in the absence of honeydew, there were no positive dose–responses between the number of SLF equivalents emitted per day and the SLF caught. However, we found a negative dose–response by females to the extract alone in week 12, the last week of the season (Table 1), and a negative dose–response by males to the extract alone in weeks 9 and 12 (Table 2).

**Figure 3:**
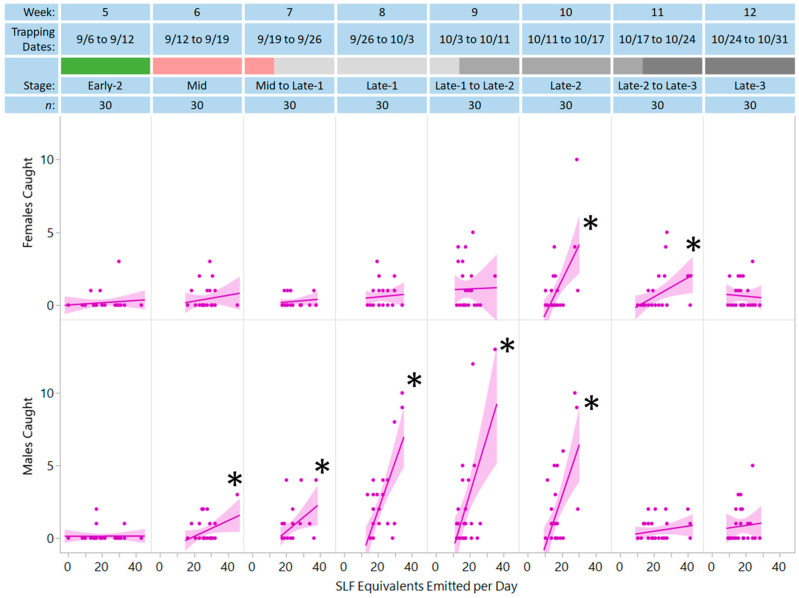
Number of females and males (y-axes) caught from week 5 onward per trap and trapping period with respect to the number of SLF equivalents emitted per day (x-axis) in the presence of honeydew. The linear regression lines and 95% confidence intervals (shaded regions) are shown, with the asterisks (*) indicating significant positive dose–responses (*p* < 0.05).

Significant positive dose–responses between the number of SLF equivalents emitted and the number of females and males captured were seen only in the presence of honeydew (Figure 3). Interestingly, when honeydew was present, males started to show a significant positive dose–response to the extract in week 6 when mating started, and the dose–response increased in weeks 7, 8, and 9, then decreased slightly in week 10, with no dose–response in weeks 11 or 12, when most mating was complete and the main activity was oviposition. Additionally, when honeydew was present, females had a positive dose–response to the extract only in weeks 10 and 11, when oviposition replaced mating as the main activity.

## 4. Discussion

This study tested natural sources of an SLF pheromone to attract conspecifics in the field, without knowing the chemical composition, blend, or ratio composing the pheromone. Two sources of possible SLF pheromones were tested—SLF honeydew and SLF whole-body extracts—each collected from a naturally occurring mixture of males and females. We found that SLF males were attracted during mating time, and only to the combination of both lures composed of naturally collected SLF pheromones from honeydew and body volatiles. When mating subsided, males were no longer attracted. This suggests that the development of a sex pheromone lure for SLF would be possible once the pheromone has been correctly identified.

Additionally, we found that females were similarly attracted to the combination of both odor sources during peak oviposition time. This result suggests the use of an aggregation pheromone by females when ovipositing. Oviposition aggregation pheromones have been reported previously in insects such as gregarious desert locusts [25] and blackflies [26], and SLF have also been found to oviposit in aggregations [4,5], making the presence of such a pheromone plausible.

Both sexes had a positive dose-dependent response to the body extract, but only in the presence of honeydew and at the appropriate times for mate-finding by males and oviposition by females. The fact that neither sex showed a positive dose–response to the body extract in the absence of honeydew further affirms the importance of honeydew volatiles in the intraspecific communication of SLF [11]. Furthermore, when honeydew was present, the dose–response to the extract signifies the importance of components from SLF body volatiles and demonstrated that, when presented together, there was a synergistic effect.

The fact that the significant positive dose–response to SLF body volatiles in the presence of SLF honeydew occurred in males during mating time suggests that semiochemicals from the combined sources are likely used by males in mate location. It also illustrates mate-finding phenology as continuing for five weeks after mating commences (until the 17th of October), with peak activity in the third, fourth, and fifth weeks after the first observation of mating. It is understood that mate-finding by males begins when we first observe mating in the field, as shown previously in both laboratory and field studies [4,7,12,22]. However, the timing of when SLF mate-finding peaks and ends was not previously well understood.

Honeydew, which is expelled away from an insect with force [27,28], likely signals the presence of feeding SLF and a suitable host, thus drawing other SLF to the vicinity, specifically below, where feeding is taking place. Subsequently, components from body volatiles are likely used by males to assist in locating female conspecifics that are ready to mate. This could explain why we only saw a positive dose–response to body volatiles while in the presence of honeydew. If the honeydew did not first attract SLF males to the correct vicinity, fewer would be present to subsequently be attracted to body volatiles. Curiously, this does not explain the two instances, during weeks 9 and 12, when males had a significant negative dose–response to the extract when honeydew was absent. Thus, a true synergism may exist between the components in both odor sources. Synergism occurs when the response to two combined stimuli is greater than the sum of responses to the individual stimuli [29].

We have previously demonstrated that SLF adult males discriminated between male and female SLF body volatiles in laboratory bioassays [12,13]. Subsequently, we described the dimorphic antennal sensitivity between male and female SLF to the volatile compounds found in honeydew and SLF body volatiles [14]. The difference between the SLF male and female volatile profiles responsible for male sexual attraction is likely attributed to qualitative differences in the presence or absence of volatile compounds and/or the ratio of the volatile compounds contained therein. A few qualitative differences between the volatiles emitted by males and females during peak mating time have been described [13], and these differences are currently under further investigation. However, no qualitative differences have been found between the honeydew of male and female SLF [11]. Since expelled honeydew is unlikely to communicate the exact location of an individual and the honeydew of males and females would mix on surfaces below feeding sites, it is likely a general indicator of the presence of a suitable host plant and feeding SLF.

In addition to demonstrating the synergism between the natural pheromone lures attracting mate-seeking males, this study revealed and informed other key aspects of SLF biology and phenology. The timing of when females started to respond and males stopped responding, during oviposition time, suggested that females were attracted to a combination of honeydew and body volatiles to locate conspecifics during oviposition. Previously, SLF aggregation during oviposition time had been documented [4,30]. This study shows that honeydew alone was not responsible for this attraction, as inferred by the dose–response to the extract while in its presence (Figure 3, Table 2). Females were attracted to the lures in the field between the 11th and 24th of October. Our previous laboratory behavioral studies were conducted before the 11th of October due to difficulties keeping SLF alive in the laboratory after this date. Thus, this was the first time we documented evidence of a possible female aggregation pheromone at the time of SLF oviposition.

A limitation of this study involves the very low number of SLF equivalents released over time by each diffuser. This limited the study area to lightly infested field sites in order to avoid having the lures compete with larger naturally occurring aggregations of SLF. Because of this, this study did not thoroughly assess attraction to pheromone lures early in the season, because so few SLF were present in the area in the first four weeks of this study that their numbers could not be analyzed. In addition, our stringent requirements for field sites to meet the SLF density, tree size, and tree spacing specifications also limited us to three trees per block, resulting in the difficult decision not to test honeydew alone. Previous laboratory studies established that the pre-mating adult stages of SLF, particularly males, were attracted to honeydew alone and body volatiles alone [11,12,13]. Even third- and fourth-instar SLF were found to be attracted to volatiles from SLF extracts in behavioral bioassays. SLF aggregation behavior occurs during both nymphal [5] and adult stages [4], and aggregations are present prior to mating. The results from this study do not touch upon how SLF form pre-mating aggregations, but they inform us about one communication mechanism used by SLF in mate location and, possibly, during oviposition.

Though the lures in this study were limited by how much insect material could be collected, the future identification and development of the pheromone components into lures would allow for higher release rates to be studied in higher-density areas, as well as testing on all mobile stages of SLF, which would enhance our understanding of their behavioral function.

Interspecific attraction to honeydew is relatively common and has been documented in various insect species. Numerous insects exploit honeydew as a food source, including, for example, house flies [31], mosquitoes [32], bees [33], wasps [34,35], and ants [36]. Some insects use honeydew volatiles to locate their honeydew-producing host or prey; thus, honeydew acts as the source of a kairomone. Examples include parasitic wasps [37,38,39], gall midges [40], lacewings, and predaceous beetles [41,42,43]. The volatiles emitted by the bacterial microbiota found in honeydew can also attract parasitoids [44,45]. However, only a few instances have been reported of honeydew components functioning as a pheromone through which conspecifics communicate their presence to one another. Such examples include psyllids [46], spittlebugs [47,48], and spotted lanternflies [11]. Interestingly, the role of honeydew has been implicated in aggregation behaviors for all of these. Our findings underscore that SLF use a complex system of pheromones requiring compounds from both honeydew and SLF body odors to locate conspecifics for mating and oviposition. Our continued efforts to understand SLF are gradually exposing their complex behavior, ecology, and communication systems, which will help guide the development of traps and lures that can be used in surveys, detection, and mitigation.

## Figures and Tables

**Table 1:** Results showing the mean number and standard error (SE) of female spotted lanternflies (SLF) *L. delicatula* captured per trapping period (with three trapping periods per week), and the results of a linear regression analysis testing for the presence of a dose–response between the number of SLF equivalents (for extract) or hexane (for controls) emitted by each lure and the corresponding number of females caught by that lure. Significant dose–responses occurred when *p* < 0.05, as shown with an asterisk. The slope indicates the intensity of the relationship and whether the response was positive or negative.

Week	Trapping Date Range	Stage	Treatment	N	Female SLF Caught	SE	Slope	R^2^	F	d.f.	*p*-Value
1	8/8 to 8/15	Early-1	Control	9	0.22	0.222	0.139	0.108	0.85	1, 7	0.388
			Extract	9	0	0	-	-	-	1, 7	-
			Extract + Honeydew	9	0.11	0.111	−0.018	0.040	0.29	1, 7	0.608
2	8/15 to 8/22	Early-1	Control	9	0.33	0.167	0	0	-	0, 8	-
			Extract	9	0	0	0	0	-	1, 7	-
			Extract + Honeydew	9	0	0	0	-	-	1, 7	-
3	8/22 to 8/29	Early-1	Control	30	0.03	0.033	−0.005	0.078	2.35	1, 28	0.136
			Extract	30	0.03	0.033	0.005	0.053	1.56	1, 28	0.222
			Extract + Honeydew	30	0	0	0	-	-	1, 28	-
4	8/29 to 9/6	Early-2	Control	30	0.17	0.084	0.013	0.083	2.52	1, 28	0.124
			Extract	30	0.10	0.056	−0.004	0.022	0.64	1, 28	0.432
			Extract + Honeydew	30	0.13	0.063	0.003	0.006	0.17	1, 28	0.680
5	9/6 to 9/12	Early-2	Control	30	0.07	0.067	0.010	0.060	1.76	1, 28	0.196
			Extract	30	0.17	0.084	0.007	0.012	0.33	1, 28	0.568
			Extract + Honeydew	30	0.17	0.108	0.008	0.012	0.33	1, 28	0.568
6	9/12 to 9/19	Mid	Control	30	0.23	0.092	0.002	0.001	0.02	1, 28	0.895
			Extract	30	0.20	0.088	−0.011	0.014	0.39	1, 28	0.538
			Extract + Honeydew	30	0.40	0.141	0.020	0.021	0.60	1, 28	0.447
7	9/19 to 9/26	Mid-to-Late-1	Control	30	0.40	0.113	0.020	0.070	2.11	1, 28	0.157
			Extract	30	0.37	0.131	−0.040	0.111	3.52	1, 28	0.071
			Extract + Honeydew	30	0.23	0.079	0.001	0.015	0.43	1, 28	0.518
8	9/26 to 10/3	Late-1	Control	30	0.30	0.119	0.007	0.003	0.08	1, 28	0.778
			Extract	30	0.40	0.132	−0.009	0.004	0.11	1, 28	0.745
			Extract + Honeydew	30	0.57	0.141	0.011	0.007	0.19	1, 28	0.667
9	10/3 to 10/11	Late-1-to-Late-2	Control	30	0.70	0.145	−0.051	0.065	1.96	1, 28	0.172
			Extract	30	0.50	0.115	−0.016	0.012	0.33	1, 28	0.572
			Extract + Honeydew	30	1.10	0.277	0.005	0.000	0.01	1, 28	0.931
10	10/11 to 10/17	Late-2	Control	30	0.63	0.282	0.097	0.093	2.88	1, 28	0.101
			Extract	30	0.70	0.160	−0.032	0.017	0.49	1, 28	0.489
			Extract + Honeydew	30	0.93	0.371	0.239	0.307	12.43	1, 28	0.002 *
11	10/17 to 10/24	Late-2-to-Late-3	Control	30	0.70	0.268	0.045	0.027	0.79	1, 28	0.382
			Extract	30	0.80	0.246	0.006	0.001	0.04	1, 28	0.839
			Extract + Honeydew	30	0.70	0.232	0.066	0.180	6.15	1, 28	0.019 *
12	10/24 to 10/31	Late-3	Control	30	1.77	0.660	0.280	0.063	1.89	1, 28	0.181
			Extract	30	1.63	0.344	−0.100	0.088	2.71	1, 28	0.005 *
			Extract + Honeydew	30	0.63	0.162	−0.010	0.004	0.11	1, 28	0.749

**Table 2:** Results showing the mean number and standard error (SE) of male spotted lanternflies (SLF) *L. delicatula* captured per trapping period (with three trapping periods per week), and the results of a linear regression analysis testing for the presence of a dose–response between the number of SLF equivalents (for extract) or hexane (for controls) emitted by each lure and the corresponding number of males caught by that lure. Significant dose–responses occurred when *p* < 0.05, as shown with an asterisk. The slope indicates the intensity of the relationship and whether the response was positive or negative.

Week	Trapping Date Range	Stage	Treatment	N	Male SLF Caught	SE	Slope	R^2^	F	d.f.	*p*-Value
1	8/8 to 8/15	Early-1	Control	9	0.11	0.111	0.070	0.108	0.85	1, 7	0.388
			Extract	9	0.11	0.111	−0.022	0.067	0.50	1, 7	0.502
			Extract + Honeydew	9	0	0	0	-	-	1, 7	-
2	8/15 to 8/22	Early-1	Control	9	0	0	0	-	-	0, 8	-
			Extract	9	0.22	0.147	0.061	0.139	1.13	1, 7	0.324
			Extract + Honeydew	9	0	0	0	-	-	1, 7	-
3	8/22 to 8/29	Early-1	Control	30	0.10	0.056	−0.002	0.006	0.17	1, 28	0.682
			Extract	30	0	0	0	-	-	1, 28	-
			Extract + Honeydew	30	0.03	0.033	−0.006	0.029	0.83	1, 28	0.369
4	8/29 to 9/6	Early-2	Control	30	0.07	0.046	0.004	0.021	0.61	1, 28	0.442
			Extract	30	0	0	0	-	-	1, 28	-
			Extract + Honeydew	30	0.03	0.033	−0.004	0.033	0.94	1, 28	0.340
5	9/6 to 9/12	Early-2	Control	30	0.10	0.056	0.000	0.000	0.00	1, 28	0.991
			Extract	30	0.13	0.080	0.003	0.003	0.08	1, 28	0.786
			Extract + Honeydew	30	0.13	0.080	0.001	0.000	0.01	1, 28	0.917
6	9/12 to 9/19	Mid	Control	30	0.63	0.256	0.000	0.000	0.00	1, 28	0.952
			Extract	30	0.13	0.079	0.002	0.000	0.01	1, 28	0.917
			Extract + Honeydew	30	0.46	0.150	0.054	0.131	4.23	1, 28	0.049 *
7	9/19 to 9/26	Mid-to-Late-1	Control	30	0.87	0.234	0.015	0.010	0.27	1, 28	0.608
			Extract	30	0.87	0.248	−0.045	0.039	1.14	1, 28	0.294
			Extract + Honeydew	30	0.70	0.226	0.097	0.174	5.91	1, 28	0.022 *
8	9/26 to 10/3	Late-1	Control	30	1.60	0.433	0.136	0.094	2.89	1, 28	0.100
			Extract	30	1.07	0.299	0.035	0.012	0.34	1, 28	0.565
			Extract + Honeydew	30	2.10	0.497	0.331	0.485	26.40	1, 28	<0.001 *
9	10/3 to 10/11	Late-1-to-Late-2	Control	30	2.30	0.484	−0.087	0.017	0.49	1, 28	0.493
			Extract	30	0.90	0.188	−0.104	0.184	6.31	1, 28	0.018 *
			Extract + Honeydew	30	2.20	0.598	0.385	0.322	13.31	1, 28	0.001 *
10	10/11 to 10/17	Late-2	Control	30	0.90	0.218	−0.025	0.010	0.30	1, 28	0.591
			Extract	30	1.80	0.390	0.174	0.087	2.67	1, 28	0.114
			Extract + Honeydew	30	1.70	0.500	0.349	0.365	16.09	1, 28	<0.001 *
11	10/17 to 10/24	Late-2-to-Late-3	Control	30	0.57	0.196	0.001	0.002	0.06	1, 28	0.806
			Extract	30	0.73	0.283	0.015	0.007	0.206	1, 28	0.654
			Extract + Honeydew	30	0.50	0.133	0.017	0.038	1.11	1, 28	0.301
12	10/24 to 10/31	Late-3	Control	30	1.90	0.700	0.265	0.050	1.48	1, 28	0.233
			Extract	30	1.20	0.240	−0.104	0.194	6.77	1, 28	0.015 *
			Extract + Honeydew	30	0.83	0.235	0.017	0.005	0.13	1, 28	0.719

## Data Availability

The raw data supporting the conclusions of this article will be made available by the authors, without undue reservation.

## Supplementary Materials

The following supporting information can be downloaded at https://www.mdpi.com/article/10.3390/insects15060447/s1, Figure S1: Number of females and males (y-axes) caught from week 5 onward per trap and trapping period with respect to the number of hypothetical SLF equivalents emitted per day (x-axis) from control hexane lures in the absence of honeydew. Linear regression lines and 95% confidence intervals (shaded regions) are shown. No significance was found; and Figure S2: Number of females and males (y-axes) caught from week 5 onward per trap and trapping period with respect to the number of SLF equivalents emitted per day (x-axis) from extract lures in the absence of honeydew. Linear regression lines and 95% confidence intervals (shaded regions) are shown, with asterisks indicating significant negative dose–responses (*p* < 0.05).
