# Supplementary material for: Spotted Lanternflies Respond to Natural Pheromone Lures for Mate-Finding and Oviposition
Source: Insects. 2024 Jun 13;15(6):447. doi: 10.3390/insects15060447 (PMC11203839; doi:10.3390/insects15060447)
Supplement: Supplementary file 1 [file insects-15-00447-s001.zip › insects-3062515-supplementary.pdf]

Figure S1

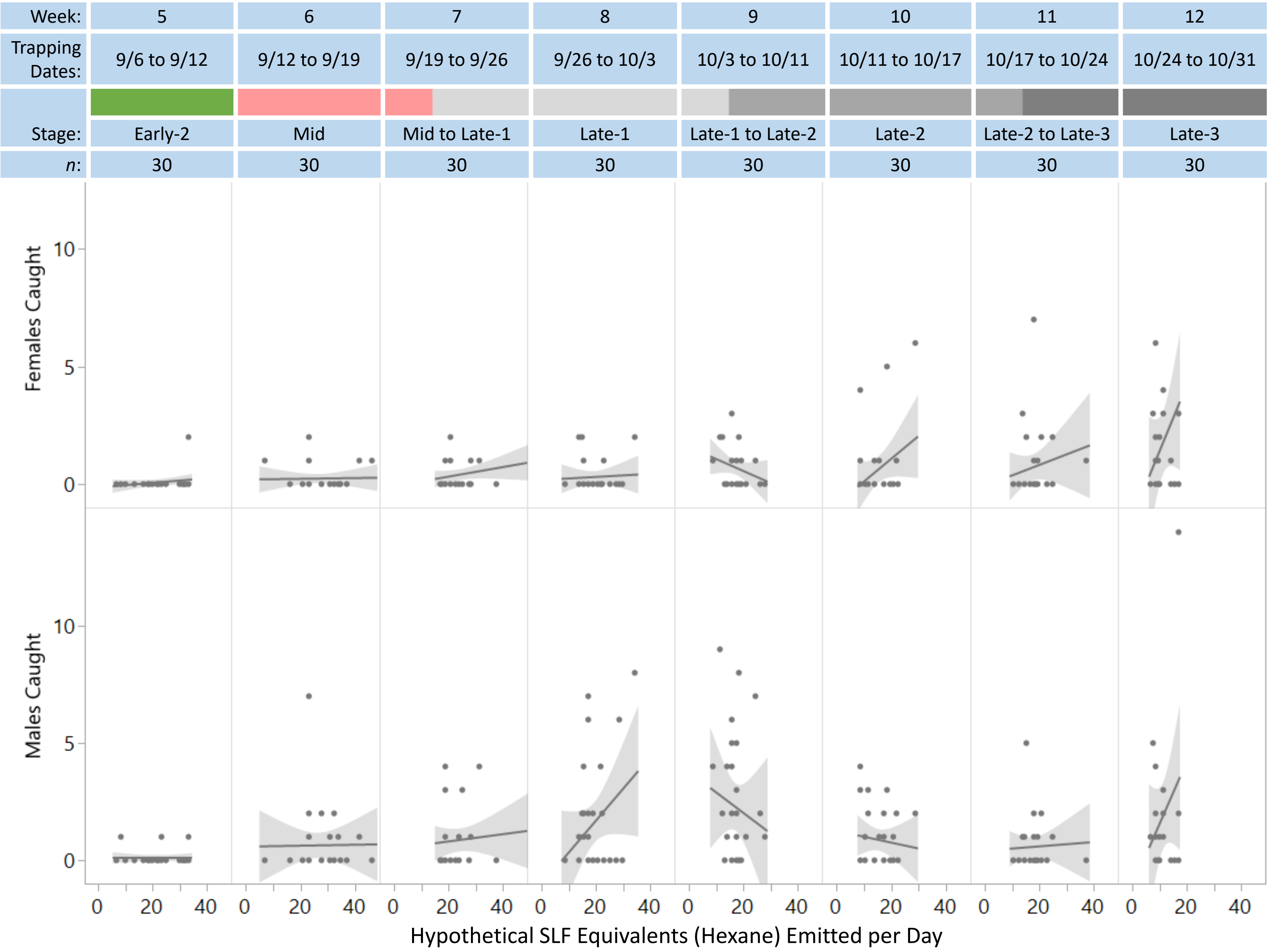

Figure S1. Number of females and males (y-axes) caught from week 5 onward per trap and trapping period with respect to the number of hypothetical SLF equivalents emitted per day (x-axis) from control hexane lures, in the absence of honeydew. Linear regression lines and 95% confidence intervals (shaded regions) are shown. No significance was found.

Figure S2

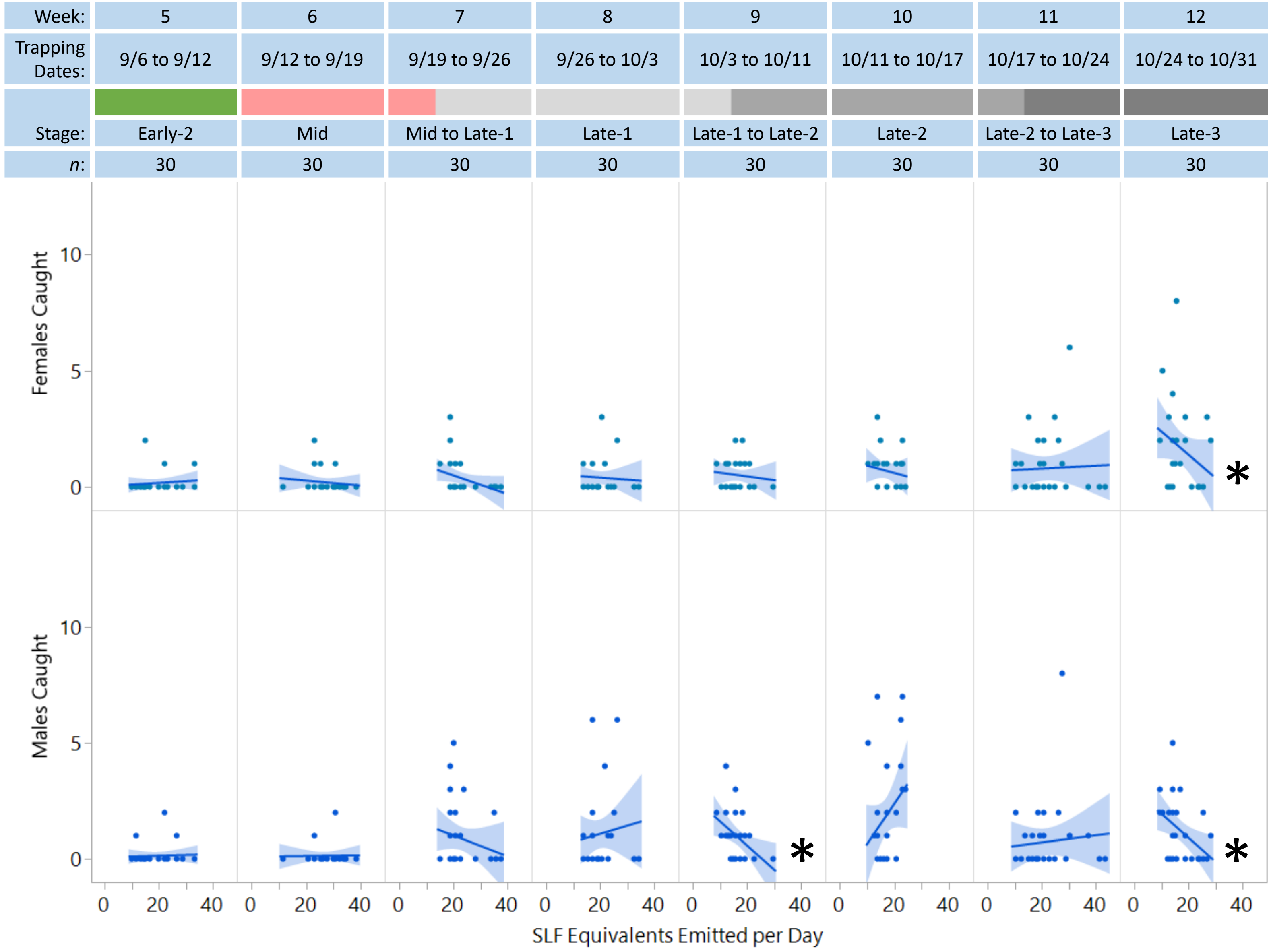

Figure S2. Number of females and males (y-axes) caught from week 5 onward per trap and trapping period with respect to the number of SLF equivalents emitted per day (x-axis) from extract lures in the absence of honeydew. Linear regression lines and 95% confidence intervals (shaded regions) are shown, with asterisks indicating significant negative dose-responses (*P<0.05*).
